# Co-regulation and multilocus determinants of gene expression in humans

**DOI:** 10.1186/1753-6561-1-s1-s88

**Published:** 2007-12-18

**Authors:** Berit Kerner, Julia N Bailey, Rita M Cantor

**Affiliations:** 1 Center for Neurobehavioral Genetics, Department of Psychiatry and Biobehavioral Sciences, Semel Institute for Neuroscience and Human Behavior, 695 Charles E. Young Drive South, Los Angeles, California 90095-1761, USA; 2 Center for Primate Neuroethology, Department of Psychiatry and Biobehavioral Sciences, Semel Institute for Neuroscience and Human Behavior, 695 Charles E. Young Drive South, Los Angeles, California 90095-1761, USA; 3 Departments of Human Genetics and Pediatrics, David Geffen School of Medicine at UCLA, 695 Charles E. Young Drive South, Los Angeles, California 90095-7088, USA

## Abstract

**Background:**

The regulation of gene expression is an emerging area of investigation. Increased knowledge can deepen our understanding of the genetic contributions to variations in complex traits. The purpose of this study is to explore the feasibility of detecting regulatory elements of gene expression with multivariate analyses.

**Methods:**

Peripheral blood lymphocyte expression levels of 30 genes on chromosome 5 and a single gene, *DEAD*, on chromosome 22 were analyzed in single-point variance-component linkage analyses in multiplex families to identify putative regulatory regions. To explore the possibility of regulatory regions having individual relationships with the expression levels of a single gene, we utilized stepwise regression. To explore the possibility of pleiotropy of a single regulatory locus for multiple genes, bivariate linkage analysis was applied.

**Results:**

Twenty-one loci were linked to five expression levels. The two most significant were for the known region on chromosome 22 (LOD = 4.62). On chromosome 5 a LOD of 4.57 was found for the gene *leukocyte-derived arginine aminopeptidase (LRAP) *with a single-nucleotide polymorphism (SNP) within 5 Mb. Both genes showed evidence of linkage to multiple SNPs. When 194 family members were treated as independent, stepwise regression identified fewer single-nucleotide polymorphisms with significant predictive values (*p *< 0.05), providing evidence for multiple regulatory regions of unequal effect. However, when corrections for non-independence were applied these results could no longer be detected.

**Conclusion:**

The complex nature of gene regulation can be explored by linkage analysis with single-nucleotide polymorphisms followed by multivariate methods to explore co-regulation.

## Background

Regulation of gene expression in human peripheral blood lymphocytes is not well understood. Most knowledge comes from the study of individual genes and their nearby regulatory elements. Taking this approach, a relatively small number of differentially expressed genes has been identified, and their regulatory elements found to have a major effect on gene expression (>50%).

However, emerging evidence indicates that gene expression is regulated by a network of elements that are both close to the gene and at large distances. Studies in flies, yeast, and mice suggest that most genes are regulated by many regulatory elements with small effects and these complicated interactions may be difficult to detect with current methods of analysis [1]. High-throughput methods for single-nucleotide polymorphism (SNP) genotyping and detection of expression levels allow us to further investigate these mechanisms.

We report a study designed to explore the feasibility of detecting regulatory elements of gene expression with multivariate analyses.

## Methods

### The Genetic Analysis Workshop 15 (GAW15) sample

Gene expression data from lymphoblastoid cell lines of 194 individuals from 14 three-generation Centre d'Etude du Polymorphisme Humain families obtained using an Affimetrix Human Focus Array were provided. This study was approved by the Institutional Review Board at UCLA.

### Selection of genes and SNPs

To apply our method we focused our analysis on 90 gene expression traits for those genes residing on chromosome 5q. One trait on chromosome 22 with a known significant linkage signal to a SNP in close proximity to the gene was selected as well [2]. For these genes, we tested the normality of expression levels, and estimated their heritability using the SOLAR software [3]. We also included sex as a covariate in the genetic model to test its significance for the expression levels of the genes. Only traits with a heritability ≥ 0.2 and skewness and kurtosis estimates within the normal range were retained. Thirty traits on chromosome 5q and chromosome 22 met these criteria. Four SNPs on chromosome 5 had a Mendelian error in two families and eight SNPs on chromosome 22 had Mendelian errors in four families. Those families with genotypes with Mendelian inconsistencies were eliminated for the particular SNP.

### Linkage analysis

Single and bivariate trait single-point variance-component (VC) quantitative trait linkage (QTL) analyses were performed using the SOLAR software on gene expression levels for the 31 genes selected. Given the relatively small number of pedigrees and the exploratory nature of these studies, a LOD of 2.0 was used as the threshold for linkage.

### Multivariate analyses

In cases in which multiple SNPs were linked to an expression trait, stepwise multivariate regression analysis was used to identify loci that were the strongest predictors of the expression levels, and to understand if all loci contributed equally [4]. In stepwise regression the SNP genotypes for each individual were used as explanatory variables after recoding them in a co-dominant fashion. Sex was included in the analysis to determine its possible significance. Analyses were conducted using SAS software version 9.1 [5]. A significance level of 0.05 was set as a criterion for entry into the model, as well as for remaining in the model. Because our data were collected in families that shared genetic information, they were not uncorrelated. We therefore took non-independence of the observations into account by using the final model from the stepwise regression and calculating a robust standard error using Proc Surveyreg. This procedure computes the regression coefficient estimation by generalized least-squares estimation using element-wise regression and the Taylor expansion theory for estimating sampling errors of estimation based on complex sample designs [5].

### Test for pleiotropy

To test for the possibility that a single gene (allele) influences the expression levels of multiple traits, we extended the univariate genetic analyses to a bivariate analysis in which the bivariate phenotypes are modeled as the outcome and genetic and environmental correlations can be estimated as described in detail by Almasy et al. [6]. A model in which all parameters are estimated is compared with a model in which the genetic correlation is constrained to zero to test for pleiotropic effects. The significance of this test implies a common set of genes contributing to the variance in the two traits. This model can be extended to incorporate SNP data. We can then test for linkage by comparing likelihoods of the model with and without the genetic data. Twice the difference of the log likelihoods of the two models is distributed asymptotically as a chi-square with 1 degree of freedom. A bivariate analysis was conducted in SOLAR for those genes where expression levels showed evidence of co-regulation by a single common SNP in order to test for the possibility that such a joint analysis would increase the power to detect linkage.

## Results

### Linkage and multivariate analysis

Out of the 30 heritable expression level traits on chromosome 5, 4 showed evidence of linkage at a LOD score > 2 to 11 SNPs on the same chromosome (Table 1). The heritability estimates of these traits ranged from 0.41 to 0.61 and sex as a covariate did not have a significant effect. The strongest linkage signal (LOD score of 4.6) was obtained between variation in expression levels of the gene *LRAP *and the SNP rs18224780, located approximately 5 megabase pairs (Mb) away from the gene locus.

**Table 1:** Expression traits with linkage to SNPs on Chromosomes 5 and 22

Expression Trait	Trait name	Chr	Location (Mb)	Heritability	LOD score	SNP	Location (Mb)
*LRAP*	Leukocyte-derived arginine aminopeptidase	5q15	96.2	0.59	**4.6**	**rs1824780**^a^	**99.7**
					3.4	rs1973172	99.7
					3.1	rs1824777	99.6
					2.8	rs421508	83.3
					2.8	rs1464765	111.6
					2.3	rs1020720	80.5
							
*SAR1B*	SAR1 gene homolog B (*S. cerevisiae*)	5q31.1	133.9	0.45	2.1	rs428538	104
							
*TCOF1*	Treacher Collins-Franceschetti syndrome 1	5q32-33	149.7	0.41	2.3	rs984091	66.5
							
*GRK6*	G protein-coupled receptor kinase 6	5q35	176.7	0.61	2.8	rs984091	66.5
					2.5	rs334908	34.7
					2.2	rs506056	39.8
							
*DDX17*	DEAD (Asp-Gl-Ala-Asp) box polypeptide 17	22q13	37.2	0.59	4.6	rs132404	43.4
					**4.1**	**rs80533**	**39.4**
					3.7	rs141418	32.8
					3.5	rs738733	46.6
					**3.3**	**rs760482**	**37.5**
					3	rs734139	35.9
					2.8	rs728591	46.5
					2.3	rs916336	34.9
					2.2	rs2032474	32
					2	rs713912	42.3

^a^Bold text indicates SNPs that remained in the stepwise regression analyses.

Expression levels of two genes on chromosome 5 showed evidence of linkage to multiple SNPs (Table 1), and in these cases, stepwise regression was used to select the most significant predictors of expression. For the gene *LRAP*, the SNP rs1824780 was significant at the 0.05 level, however, when we took the non-independence of the observations into account by using the final model from the stepwise regression and calculating robust standard errors using Proc Surveyreg, those SNPs were no longer significant (Table 2). The second gene, G protein-coupled receptor kinase 6 (*GRK6*), did not reveal significant results in stepwise regression.

**Table 2:** Results of stepwise regression

Gene	SNP	F-Value	Pr > F
*LARP*	rs1824780	4.93	0.03
			
*DDX*17	rs760482	9.23	0.003
	rs80533	4.02	0.046

**Figure 1:**
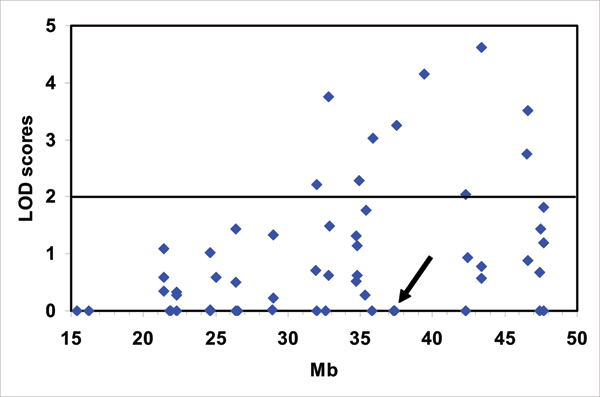
**Single-point QTL analysis for the trait gene expression level of the gene *DEAD *(*DDX17*)**. The horizontal axis shows the distance along chromosome 22 in megabase pairs (Mb). The vertical axis shows the LOD scores. The gene itself, located on chromosome 22q13 at 37.2 Mb, is indicated by the arrow.

The gene DEAD (*DDX17*), located on chromosome 22, gave multiple strong linkage signals along the entire long arm of chromosome 22 (Figure 1, Table 1). The highest LOD score (LOD = 4.62 at SNP rs132404) was located approximately 9 Mb away from the location of the gene. We included all SNPs with evidence for linkage (LOD > 2) in a stepwise regression analysis (Table 2). Only two of these SNPs (rs760482 and rs80533) remained in the model at *p *< 0.05. These SNPs were located within 5 Mb of the gene locus. None of these three genes had significant sex effects in the stepwise regression analysis.

### Pleiotropy

**Figure 2:**
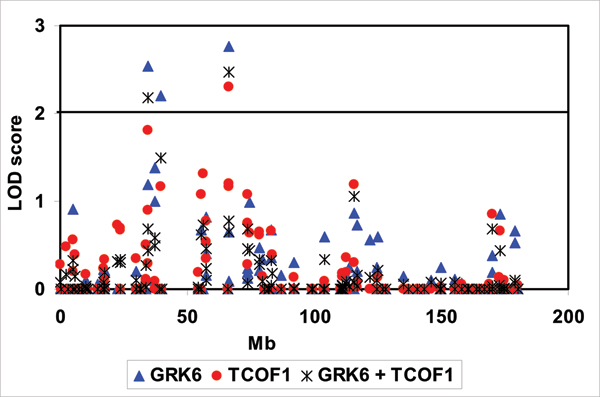
**Univariate and bivariate single-point QTL analyses for the traits gene expression levels of the genes *GRK6 *and *TCOF1 *along chromosome 5**. Gene expression levels of the genes *TCOF1 *and *GRK6 *are linked to the same SNPs. *, joint single-point QTL analysis. The distance along chromosome 5 in megabase pairs (Mb) is on the horizontal axis. LOD scores of QTL analyses are on the vertical axis.

The results of the combined polygenic VC linkage analysis showed that the genes Treacher Collins-Franceschetti syndrome 1 (*TCOF1*) and *GRK6 *share a strong and significant genetic correlation (ρ_g _= 0.94 ± 0.07, *p *< 0.0001), implying these two expression traits may share susceptibility genes at that locus, and that these two expression traits may be regulated by the same SNP. The environmental correlation for these expression levels (ρ_e _= 0.58 ± 0.09, *p *= 0.00002) was also significant. Single-point linkage analysis, which includes a major gene for both traits jointly, showed slight improvement in the LOD score at the linked SNP for trait *TCOF1 *(LOD = 2.3 for the trait alone compared to LOD = 2.5 in the joint analysis) and a slight decrease in the LOD score for trait *GRK6 *(LOD = 2.8 compared to LOD = 2.5 in the joint analysis) (Figure 2). Thus, we have no evidence that a single SNP contributes to the expression levels of these two genes.

## Discussion

In an exploratory linkage study of 31 expression traits on chromosomes 5 and 22, we found significant linkage signals for two traits, *LRAP *and *DDX17*, with SNPs in close proximity to the gene loci (*cis*). Sex as a covariate was not significant in our analysis. Other GAW15 groups who analyzed these data reported similar results [7,8]. Those two expression traits also showed evidence of linkage to multiple SNPs at considerable distances away from the gene locations (*trans*). In an evaluation of co-regulation, the SNPs did not remain significant in a stepwise regression when the non-independence of the data due to the family structure was taken into account. Evidence for potential pleiotropy was not supported by bivariate QTL analysis. Unfortunately, this study is limited by the number of families we could include in the analyses.

## Conclusion

We demonstrate here that the complex nature of gene regulation can be explored by linkage analysis with SNPs followed by multivariate methods to explore co-regulation.

## Competing interests

The author(s) declare that they have no competing interests.

## References

[B1] Stamatoyannopoulos JA (2004). The genomics of gene expression. Genomics.

[B2] Morley M, Molony CM, Weber T, Devlin JL, Ewens KG, Spielman RS, Cheung VG (2004). Genetic analysis of genome-wide variation in human gene expression. Nature.

[B3] Almasy L, Blangero J (1998). Multipoint quantitative trait linkage analysis in general pedigrees. Am J Hum Genet.

[B4] Hocking RR (1976). The analysis and selection of variables in linear regression. Biometrics.

[B5] SAS Institute Inc (2004). SAS Online Documentation, Version 91.

[B6] Almasy L, Dyer TD, Blangero J (1997). Bivariate quantitative trait linkage analysis: pleiotropy versus co-incident linkages. Genet Epidemiol.

[B7] Sung YJ, Di Y, Fu AQ, Rothstein JH, Sieh W, Tong L, Thompson EA, Wijsman EM (2007). Comparison of multipoint linkage analyses for quantitative traits in the CEPH data: parametric LOD scores, variance components LOD scores, and Bayes factors. BMC Proc.

[B8] Rangrej J, Beyene J, Hu P, Paterson AD (2007). Sex, age, and generation effects on genome-wide linkage analysis of gene expression in transformed lymphoblasts. BMC Proc.

